# Evaluation of Telemedicine Consultations Using Health Outcomes and User Attitudes and Experiences: Scoping Review

**DOI:** 10.2196/53266

**Published:** 2024-07-09

**Authors:** Pranavsingh Dhunnoo, Bridie Kemp, Karen McGuigan, Bertalan Meskó, Vicky O’Rourke, Michael McCann

**Affiliations:** 1 Department of Computing Atlantic Technological University Letterkenny Ireland; 2 The Medical Futurist Institute Budapest Hungary; 3 School of Nursing and Midwifery Queen's University Belfast Belfast United Kingdom; 4 Faculty of Business Atlantic Technological University Letterkenny Ireland

**Keywords:** telemedicine, internet-based consultation, chronic illnesses, cyberpsychology, digital health, scoping review

## Abstract

**Background:**

Despite a recent rise in adoption, telemedicine consultations retention remains challenging, and aspects around the associated experiences and outcomes remain unclear. The need to further investigate these aspects was a motivating factor for conducting this scoping review.

**Objective:**

With a focus on synchronous telemedicine consultations between patients with nonmalignant chronic illnesses and health care professionals (HCPs), this scoping review aimed to gain insights into (1) the available evidence on telemedicine consultations to improve health outcomes for patients, (2) the associated behaviors and attitudes of patients and HCPs, and (3) how supplemental technology can assist in remote consultations.

**Methods:**

PRISMA-ScR (Preferred Reporting Items for Systematic Reviews and Meta-Analyses extension for Scoping Reviews) guided the scoping review process. Inclusion criteria were (1) involving adults with nonmalignant, noncommunicable chronic conditions as the study population; (2) focusing on health outcomes and experiences of and attitudes toward synchronous telemedicine consultations between patients and HCPs; and (3) conducting empirical research. A search strategy was applied to PubMed (including MEDLINE), CINAHL Complete, APA PsycNet, Web of Science, IEEE, and ACM Digital. Screening of articles and data extraction from included articles were performed in parallel and independently by 2 researchers, who corroborated their findings and resolved any conflicts.

**Results:**

Overall, 4167 unique articles were identified from the databases searched. Following multilayer filtration, 19 (0.46%) studies fulfilled the inclusion criteria for data extraction. They investigated 6 nonmalignant chronic conditions, namely chronic obstructive pulmonary disease, diabetes, chronic kidney disease, ulcerative colitis, hypertension, and congestive heart failure, and the telemedicine consultation modality varied in each case. Most observed positive health outcomes for patients with chronic conditions using telemedicine consultations. Patients generally favored the modality’s convenience, but concerns were highlighted around cost, practical logistics, and thoroughness of clinical examinations. The majority of HCPs were also in favor of the technology, but a minority experienced reduced job satisfaction. Supplemental technological assistance was identified in relation to technical considerations, improved remote workflow, and training in remote care use.

**Conclusions:**

For patients with noncommunicable chronic conditions, telemedicine consultations are generally associated with positive health outcomes that are either directly or indirectly related to their ailment, but sustained improvements remain unclear. These modalities also indicate the potential to empower such patients to better manage their condition. HCPs and patients tend to be satisfied with remote care experience, and most are receptive to the modality as an option. Assistance from supplemental technologies mostly resides in addressing technical issues, and additional modules could be integrated to address challenges relevant to patients and HCPs. However, positive outcomes and attitudes toward the modality might not apply to all cases, indicating that telemedicine consultations are more appropriate as options rather than replacements of in-person visits.

## Introduction

### Background

While the underpinning technology has been available for several decades [1], telemedicine consultations experienced a surge in adoption following the COVID-19 pandemic [2]. In particular, for patients with chronic conditions, these modes of care lessen travel-associated pain as well as facilitate the management and treatment of their disease [3,4]. Such technology-mediated consultations are also favored by physicians treating patients with chronic conditions, as they can improve productivity, patient’s health, and management [4]. Continued interest in telemedicine consultation from researchers, investors, and policy makers indicates that such modalities are being actively considered as viable options within health care systems beyond the pandemic [5-9].

However, persisting issues have been raised regarding aspects such as privacy, reliability, safety, and accessibility when using remote consultation means [10-13]. Due to difficulties in identifying nonverbal cues through such means, Kilvert et al [14] recommend that the first meeting between the health care professional (HCP) and the patient be conducted face to face rather than through internet-based means, suggesting that the latter approach is not totally apt to fully replace physical meetings. The scoping review by Leone et al [15] also identified discrepancies in telehealth consultation guidelines, which indicate that the modality is still nascent and evolving.

Recent studies also reflect a need to better understand telemedicine consultation adoption by patients with chronic conditions [16,17]. Notably, a 2021 analysis by McKinsey & Company identified a stabilized use of telehealth following an initial peak early in the pandemic as telehealth was borne out of necessity [18]. Even if the use of the modality is higher than prepandemic levels, only approximately 40% of responders in the United States would be inclined to use telehealth after the pandemic subsides. However, 40% to 60% would be interested in internet-based health care with more options, such as a *digital front door* or a lower-cost virtual-first health plan. While this analysis might be more representative of the US market, it nevertheless indicates the potential for supplemental technologies to telehealth for addressing flailing retention rates and barriers to adoption.

Such challenges to maintaining and encouraging remote care use highlight the importance of gaining a deeper understanding of the technical and psychological elements, that is, cyberpsychological elements, involved in telemedicine consultations, as well as indicate potentials for exploring how novel technological approaches can effectively tackle them [4]. Incorporating supplementary technologies presents an opportunity for innovation and for transforming the adoption of web-based modalities for patients with chronic conditions. For instance, the study by Yuan et al [19] on the remote care outcomes of patients with heart failure during the pandemic found that telephone consultations were associated with increased mortality. By supplementing this modality with other technologies, there is a possibility of achieving better outcomes. Researchers further consider the metaverse and enabling technologies such as extended reality as potential approaches to supplement remote care [20]. These approaches represent potential novel avenues to consider in telemedicine consultation research.

While recent reviews have analyzed the state of telemedicine consultations from the perspective of either those living with chronic conditions [21-23] or HCPs [24], they did not investigate extensively, within the same review, perspectives of both HCPs and patients with chronic conditions and their associated behaviors or potentials for technological improvements during synchronous telemedicine consultations. Furthermore, a preliminary search for existing scoping reviews and systematic reviews performed in April 2023 on Google Scholar and Open Science Framework did not identify such investigations. Researchers have recently accentuated the need to better understand the preferences and concerns of remote health users [25]. They have also recommended further exploration of technological assistance to reduce limitations experienced during telemedicine consultations [4]. These reflect the conceptual framework developed by Hensel et al [26] that highlights the importance of investigating aspects of cyberpsychological obtrusiveness and the adoption of telehealth [26]. However, such aspects have not been widely investigated in recent reviews and indicate the need for new research in this area.

### Aims and Objectives of This Scoping Review

In addition to the potentials of telemedicine consultations, the limited availability of a synthesis of the literature in this field was a motivating factor for this scoping review, which is the first step in an on-going PhD project in Ireland at the Atlantic Technological University [27].

The project aims to develop a novel, evidence-based artificial intelligence assistive tool to supplement real-time, synchronous telemedicine consultations between patients with nonmalignant chronic illnesses and their HCPs. Considering the higher prevalence of certain chronic conditions in Ireland such as diabetes mellitus, cardiovascular conditions, and chronic obstructive pulmonary disease (COPD) [28], particular emphasis is paid on these nonmalignant conditions. Therefore, the nascent aspect of telemedicine consultations [15] and the exploratory nature of this research were deemed appropriate for the adoption of a scoping review design [29,30] that aims to investigate synchronous telemedicine consultations for nonmalignant chronic illnesses through the following research questions: (1) What is the available evidence on synchronous telemedicine consultations to improve health outcomes for patients with chronic conditions? (2) What are the associated behaviors and attitudes of patients and HCPs during synchronous telemedicine consultations? (3) How can supplemental technology assist in remote consultations?

## Methods

This scoping review was conducted by following the PRISMA-ScR (Preferred Reporting Items for Systematic Reviews and Meta-Analyses extension for Scoping Reviews) guidelines and the methodology developed by the Joanna Briggs Institute [30,31]. The protocol for this review was also registered on Open Science Framework on April 28, 2023 [32].

### Rationale for Inclusion Criteria

The inclusion criteria were based on the Population, Concept, and Context framework, which is recommended to design relevant objectives and eligibility criteria for scoping reviews, where each component of the framework (population, concept, and context) guides the identification of the respective areas of interest of the review [33]. This framework also assisted in designing the search terms to ensure relevance to the review’s aims. Textbox 1 summarizes the inclusion and exclusion criteria of this study based on the Population, Concept, and Context framework.

**Inclusion and exclusion criteria based on Population, Concept, and Context framework for study selection.**

**Inclusion criteria**

- Population
  Adults with nonmalignant, noncommunicable chronic conditions
- Concept
  Health outcomes, experiences and attitudes toward synchronous internet-based consultations between patients and health care professionals
  Aspects around attitudes, experiences, engagement, behaviors, and intentions during such interactions
- Context
  Clinical settings and beyond
  Research centers

**Exclusion criteria**

- Population
  Patients with no or any other condition
  <18 years of age
  Malignant or communicable conditions
- Concept
  Traditional or face-to-face or non–internet-based or undefined means of consultations
  Interactions with non–health care professionals
  Asynchronous internet-based care
- Context
  Nonempirical research (eg, reviews and editorials)

Upon agreement among the authors, and with the input from a specialist librarian, the following databases and repositories were selected given their relevance to provide published as well as gray literature around the research questions: PubMed (which includes MEDLINE), CINAHL Complete, APA PsycNet, Web of Science, IEEE, and ACM Digital.

### Adopted Search Strategy

A search strategy was devised by the research team to identify sources of evidence relevant to the research aims. Terms such as “metaverse” and “mixed reality” were included given their potentials in remote care [20] and to maximize the potential to identify publications that involve novel telemedicine consultation approaches, considering the exploratory nature of this review. The search terms also included specific chronic conditions such as “diabetes mellitus” and “COPD” given their prevalence in Ireland, where subsequent stages of a project involving this scoping review will take place, and to thus provide more specific results [28].

The selected databases were searched from their inception till March 6, 2023, with no language or article type filters applied; however, for CINAHL Complete via EBSCOhost, the “Apply Equivalent Subjects” and “Suggest Subject Terms” filters were selected. The following combination of search terms was used: (“virtual consultation” OR “remote consultation” OR “telemedicine” OR “telehealth” OR “metaverse” OR “virtual reality” OR “augmented reality” OR “mixed reality” OR “extended reality”) AND (“chronic conditions” OR “chronic illnesses” OR “chronic disease” OR “diabetes mellitus” OR “chronic respiratory illnesses” OR “cardiovascular conditions” OR “obesity” OR “COPD”) AND (“attitudes” OR “experiences” OR “engagement” OR “behaviours” OR “intentions” OR “motivations” OR “psychology” OR “barriers”). Medical Subject Headings were used in CINAHL Complete, which involved the database’s recommended terms. Multimedia Appendix 1 details the latter search.

### Multilayer Filtering of Search Results

**Figure 1:**
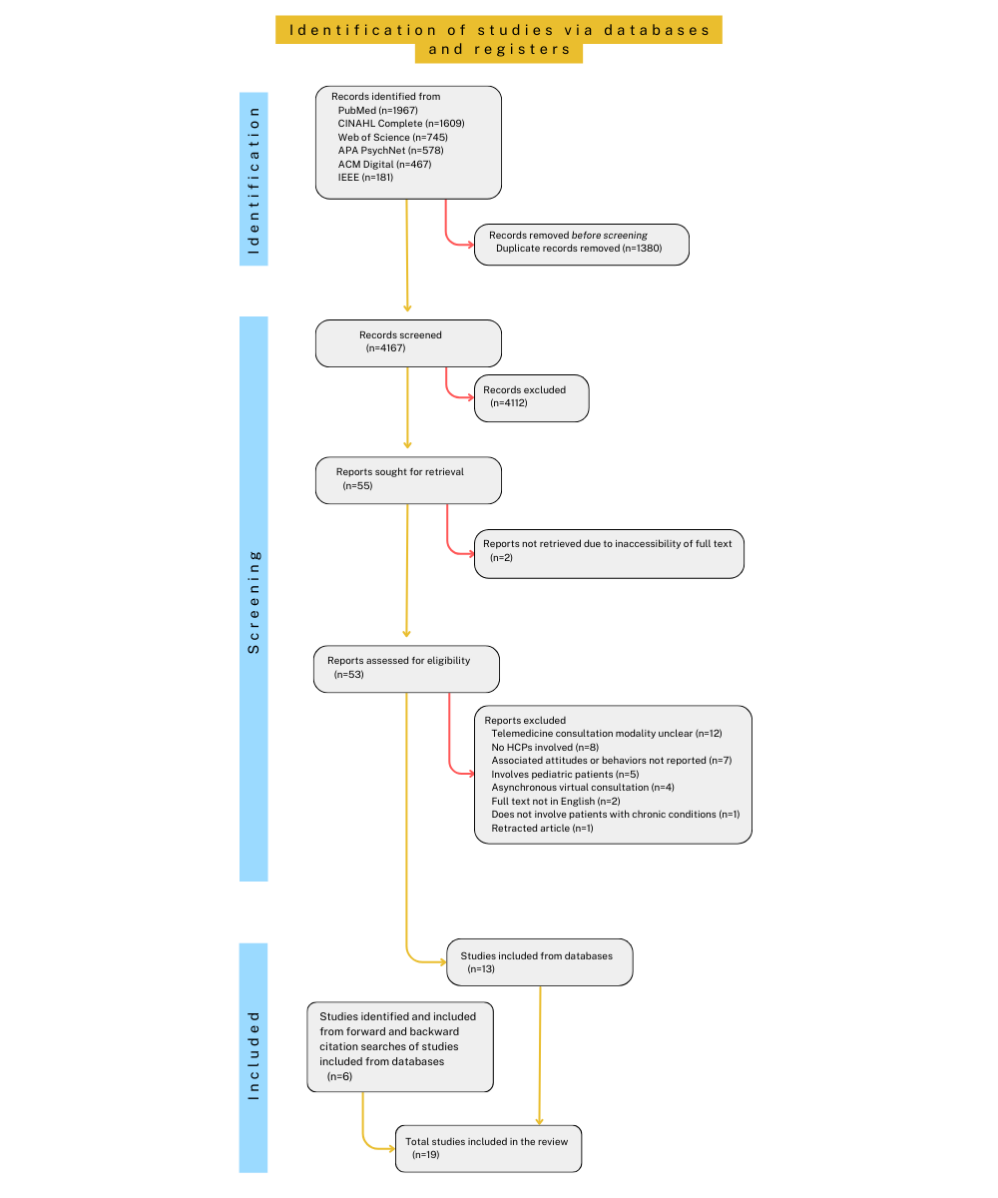
PRISMA (Preferred Reporting Items for Systematic Reviews and Meta-Analyses) 2020 flow diagram. HCP: health care professional [36].

The search results were screened independently by 2 researchers (PD and BK) via the web-based referencing software Rayyan (Rayyan Systems Inc) against the selection criteria (Textbox 1). Articles were excluded if (1) their full texts were not in English, (2) they did not involve real-time consultation with an HCP [34], (3) the (internet-based) consultation modality was unclear, (4) no consultation was involved, and (5) they were not empirical research; thus, articles such as reviews and editorials were excluded. Citation tracking was performed on publications identified from the databases whose full texts were eligible for data extraction. More specifically, backward search of their respective reference lists and forward search of articles that cite back the individual study were performed, as these are recommended practices for added value to health-related reviews [35]. After their respective screenings, the reviewers discussed and resolved any conflicts over their selection. This was done by reverting to the selection criteria and consulting with KM. The results of the screening process are summarized in Figure 1 [36], which is based on the PRISMA (Preferred Reporting Items for Systematic Reviews and Meta-Analyses) 2020 flow diagram.

Data relevant to this scoping review’s aims were independently extracted from the final list of included sources of evidence and summarized in a data-charting form by PD and KM, who resolved any conflicts after comparing their findings. This was done by reverting to the individual studies. The data-charting form was designed by the research team with items relevant to this review. Data extracted pertained to the study characteristics, including authors, year of publication, and country of origin, study design, research aims, chronic conditions involved, internet-based consultation modality used, health outcome reported by the studies, related attitudes and behaviors toward the internet-based consultation modality, and potentials for technological improvement identified in the articles.

For the synthesis of results, the included studies were grouped based on the chronic condition involved. Thereafter, data on the outcomes, type of internet-based consultation used, associated behaviors, and potentials for technological improvements were summarized. As a scoping review design was adopted for this investigation to provide an overview of current evidence relevant to the research questions, no critical appraisal of the included sources was performed, which is typical for such studies [31,37].

## Results

### Overview

Searching the databases yielded a total of 5547 articles, out of which 4167 (75.12%) unique articles were identified after the removal of duplicates. Screening of texts and abstracts against the inclusion criteria resulted in 55 (1.32%) texts for full-text screening. Of these 55 articles, 13 (24%) were found to fulfill the inclusion criteria and were selected for further data extraction and analysis. To maximize the identification of publications relevant to this review, we performed further backward and forward citation searching from these 13 articles. Such citation tracking after initial study selection is recommended practice to enhance the identification of potentially eligible reports [35]. This led to the identification of 27 additional articles for full-text screening against the inclusion criteria. This yielded 6 additional articles for inclusion in data extraction. Thus, in total, 19 studies were included for data extraction after screening 53 full texts from the database searches (of which 13 were included) and 27 full texts from backward and forward searches (of which 6 were included). The data extracted from the 19 included articles are presented in summarized form in Table 1, with more details provided in Multimedia Appendix 2 [38-56].

**Table 1:** Characteristics of sources of evidence.

Study, year, and country	Study design and aims	Chronic condition and telemedicine consultation modality	Reported health outcomes	Related attitudes and behaviors	Potentials for technological improvement
Mair et al [38], 1999, United Kingdom	Mixed method feasibility pilot of technology assistance for COPD^a^ exacerbations at home	COPD; video telemedicine consultation	Not reported	Users required getting accustomed to the setup	Need to improve image quality
Tudiver et al [39], 2007, United States	Phone survey interview to evaluate HCPs’^b^ perceptions of remote diabetes care delivery	Diabetes; video telemedicine consultation	Increased control, confidence, compliance, and motivation in managing diabetes among patients	Positivity toward telemedicine consultation	Improve communication between HCPs and remote workflow
Whitten and Mickus [40], 2007, United States	Mixed method study to evaluate the use of home telehealth for patients with COPD and CHF^c^	COPD and CHF; video telemedicine consultation	No significant effects	Satisfied with care delivery and technology	Telehealth may provide added value if the associated costs are reduced, even if the outcomes are similar to those of traditional care
Trief et al [41], 2008, United States	Qualitative study to describe the lived experiences of older adults with diabetes involved in a telemedicine management intervention	Diabetes; video telemedicine consultation	Positive physical health changes and some emotional benefit	HCPs’ encouragement can positively influence participation. Participation motivated by desire to feel and be healthier	Some frustrations with operating the technology
Nilsson et al [42], 2009, Sweden	Quantitative evaluation of the feasibility and quality of uncomplicated hypertension care in rural areas, with comparison between telemedicine and face-to-face consultations	Primary hypertension; video telemedicine consultation	Patients treated via telemedicine consultation had a higher probability of improving their blood pressure	Patients: most found telemedicine consultation to be as good as in-person GP^d^ meetings. HCPs: appreciated detailed remote patient evaluation; telemedicine consultation allowed them to work more independently	Equipment not found to be useful for remote lung examination
Cook et al [43], 2010, United States	Quantitative evaluation of remote audio nurse counseling to address cognitive and emotional barriers to medication adherence in ulcerative colitis	Ulcerative colitis; audio telemedicine consultation	Treatment adherence up to 6 months was higher than the expected rate.	Treatment discontinuation due to negative beliefs about treatment and breakthrough symptoms	Concerns around cost and treatment logistics
Sorknæs et al [44], 2010, Denmark	Mixed method interventional study to investigate the effect of telemedicine video consultations (telemedicine consultation) on early readmissions for patients with COPD in their homes after a hospital discharge	COPD; video telemedicine consultation	10% to 14% reduction in readmission risk for patients using telemedicine	High satisfaction rate with patients and nurses Nurses trusted the measurement collected from consultations.	Minimize audiovisual delay
Campbell et al [45], 2011, Canada	Mixed method feasibility study of remote care provision to rural communities and to assess the level of satisfaction among patients and HCPs	CKD^e^; video telemedicine consultation	Patients experienced less stress during remote visits than during in-person visits	High level of satisfaction The ease of access to care and time and cost savings were appreciated.	Improve the flow of paperwork and access to patient information
Berkhof et al [46], 2014, the Netherlands	Quantitative pilot study to determine the effects of telemedicine on the health care use and health status of patients with COPD	COPD; audio telemedicine consultation	No improvement of health status among the telemedicine consultation group and worsened score for the telemedicine consultation group	Use of audio telephone consultations led to little loss to follow-up	Include educational or pulmonary rehabilitation or training component to drive a more successful telemedicine model
Mathar et al [47], 2015, Denmark	Qualitative interviews investigating the telemedicine consultation experiences and preferences of patients with COPD	COPD; video telemedicine consultation	No special value attributed	Patients are receptive of the technology and to adopting at-home monitoring equipment	Interference with daily routines, cost considerations, and unwanted obligations
Raymond et al [48], 2016, United States	Pilot study to investigate the feasibility and acceptability of a telemedicine intervention	Diabetes; video telemedicine consultation	Potentials to improve mental health, diabetes education, and condition management	Satisfactory experience, given improved convenience, ease of access to HCPs’ insights, and time efficiency	None reported
Reid et al [49], 2018, United States	Quantitative cohort pilot study to assess feasibility, acceptability, care retention and follow-up rates, patient satisfaction, and adherence to guidelines in a telemedicine intervention	Diabetes; video telemedicine consultation	No significant change in HbA_1c_^f^ values in either the telemedicine consultation or control group	Higher engagement, adherence, and frequency of clinical visits in the telemedicine consultation group Higher satisfaction rates in the telemedicine consultation group regarding care experiences and technology	Barrier to access encountered due to institutional internet firewall settings
Lambooy et al [50], 2021, Australia	Quantitative case-controlled longitudinal observational cohort study to assess the feasibility, sustainability, and clinical outcomes of telehealth videoconferencing (telemedicine consultation) for patients with CKD	CKD; video telemedicine consultation	No between- group differences during follow-up for 2 years	Very high patient satisfaction with the care provided Telemedicine consultation was comparable to standard care. Telemedicine consultation uptake reduced over time.	Reduce concerns with technology and remind that telemedicine consultation is an option
Magliah et al [51], 2021, Saudi Arabia	Cross-sectional quantitative analysis of web-based surveys to evaluate patient perception of phone clinics during the COVID-19 pandemic	Diabetes; audio telemedicine consultation	Improved diabetes control and reduced risk of contracting COVID-19	Patients: satisfied with the modality; most expressed interest in remote audio follow-ups Physicians: good understanding of patients’ condition	Improve internet access
Singh et al [52], 2021, United States	Web-based surveys with quantitative analyses to determine the limitations of telehealth accessibility, patient satisfaction with telehealth relative to in-person visits, and the perceived advantages and disadvantages to telehealth	Cardiac condition; any telemedicine consultation modality	None reported	High level of satisfaction, with time and cost savings seen as advantageous Clinical examination not perceived as thorough by some patients	Improve internet connectivity, improve the coordination of telehealth appointments, and incorporate remote patient monitoring solutions
Balut et al [53], 2022, United States	Mixed methods study to understand telemedicine use before and after the COVID-19 pandemic and identify relevant barriers and facilitators to its implementation	Cardiology; audio or video visits	None reported	Patients: racial and ethnic minority groups less likely to use telemedicine; preference of telephone over video visits Physicians: preferred video to phone visits; willingness to keep using telemedicine consultation after the pandemic	Improve access to video consultations with equipment, video bandwidth, and user-friendliness of the videoconferencing platform
Heyck Lee et al [54], 2022, Canada	Quantitative cross-sectional observational survey to evaluate patients’ and physicians’ perspectives on the key advantages and disadvantages of telephone consultations in a nephrology outpatient clinic setting during the COVID-19 pandemic	CKD; audio telemedicine consultation	Reduced anxiety related to contracting COVID-19 by traveling to clinic	Patients: Comfortable with the modality Low preference for video telemedicine consultation HCPs: Reduced job satisfaction and sense of connection with patients	Improve translation and auditory feedback to increase patient access to audio consultations
Hitchcock and Heath [55], 2022, United Kingdom	Web-based questionnaire and telephone interviews to understand patients’ perspectives on telemedicine consultation and the accessibility of the service and to inform its development	Diabetes; audio or video telemedicine consultation	None reported	Appreciated at-home convenience	Increase flexibility and access to the service and team
Löhnberg et al [56], 2022, United States	Quantitative program evaluation with phone surveys to report on the transition to a remote modality for a weight management program during the COVID-19 pandemic	Obesity; audio telemedicine consultation	None reported	About half found telephone visits comparable to in-person visits. Half indicated preference for remote visits after the pandemic. Time and cost efficiency of remote care compared to in-person care favored	Video meetings for interdisciplinary team cumbersome due to hardware and software requirements; require adequate training

^a^COPD: chronic obstructive pulmonary disease.

^b^HCP: health care professional.

^c^CHF: congestive heart failure.

^d^GP: general practitioner.

^e^CKD: chronic kidney disease.

^f^HbA_1c_: hemoglobin A_1c_.

### Characteristics of Sources of Evidence

Details about the 19 included studies’ authors, origin, year of publication, aims, design, and participants can be found in Table 1. They were published between 1999 and 2022 [38-56] and conducted in 8 different countries, with 9 (47%) from the United States [39-41,43,48,49,52,53,56], 2 (11%) from the United Kingdom [38,55], 2 (11%) from Denmark [44,47], 2 (11%) from Canada [45,54], 1 (5%) from Sweden [42], 1 (5%) from the Netherlands [46], 1 (5%) from Australia [50], and 1 (5%) from Saudi Arabia [51].

Of the 19 included studies, 9 (47%) adopted a quantitative design [42,43,46,49-52,54,56], 7 (37%) adopted a mixed method design [38-40,44,45,48,53], and 3 (16%) adopted a qualitative design [41,47,55]. As each study had differing aims and scopes, the number of participants varied in each case and averaged at 401 (SD 1211.19).

Among the studies included in this review, 6 nonmalignant chronic conditions were investigated altogether, namely COPD, diabetes, chronic kidney disease, ulcerative colitis, hypertension, and congestive heart failure. However, some were more frequently investigated than others, and some studies involved more than 1 chronic condition. Of the 19 included studies, 6 (32%) involved patients with diabetes [39,41,48,49,51,55], 5 (26%) involved patients with COPD [38,40,44,46,47], 4 (21%) involved patients with chronic cardiovascular conditions (CCCs) [40,42,52,53], 3 (16%) involved patients with chronic kidney disease [45,50,54], 1 (5%) involved patients with ulcerative colitis [43], and 1 (5%) involved patients with obesity [56]. Among the 4 studies that involved CCCs, 1 (25%) involved both patients with COPD and patients with congestive heart failure [40], 1 (25%) involved patients with hypertension [42], and 2 (50%) did not specify the conditions but involved patients with unspecified CCCs [52,53].

The remote consultation modality varied among the 19 included studies, with 12 (63%) using only videoconferencing [38-42,44,45,47-50,55] and 5 (26%) using only telephone visits [43,46,51,54,56]. Furthermore, 1 (5%) study considered all telehealth modalities [52], and 1 (5%) study involved both telephone and video visits [53]. Among the 12 included studies that were published before the COVID-19 pandemic [38-49], the vast majority (10/12, 83%) used video consultations [38-42,44,45,47-49], while only 2 (17%) used only telephone visits [43,46]. In comparison, among the 7 included studies that were published after the pandemic [50-56], the telemedicine consultation modality was more diverse, with 3 (43%) studies involving telephone consultations only [51,54,56], 2 (29%) involving video consultations only [50,55], 1 (14%) involving both video and telephone means [53], and 1 (14%) considering any telemedicine consultation modality [52].

### Reported Health Outcomes

The majority of the included studies (14/19, 74%) reported the health outcomes of the patients undertaking internet-based consultations, while 5 (26%) did not provide such data due to different scopes of their research aims [38,52,53,55,56]. Of the reviewed articles that reported on these, most (n=9, 64%) observed positive health outcomes for patients with chronic conditions using telemedicine consultation modalities [39,41-45,48,51,54]. These modes of care delivery were associated with better condition management [39,51], improved physiological parameters [41,42], higher treatment adherence [43], and reduced risk of readmission [44]. Remote visits also positively impacted patients’ mental health, with reported reduction in anxiety [45,48,54]. Some articles found no notable improvements or particular contribution to addressing health-related issues when using telemedicine consultations [40,46,47,49,50]. Of the 19 included studies, 1 (5%) even found deteriorating symptoms among patients in the telemedicine group based on the adopted Clinical COPD Questionnaire scale [46].

Diabetes and COPD were the most commonly investigated conditions in the included studies with reported health outcomes. Patients with diabetes generally experienced positive health outcomes relating to their physical and mental health as well as the management of their condition [39,41,48,50], while a minority experienced no changes compared to in-person consultation [49]. In the case of patients with COPD, most of the included studies with reported health outcomes indicated no additional health benefits via telemedicine consultations [40,46,47], while only a minority associated telemedicine consultations with positive health outcomes [44]. However, despite technological improvements over the timeline of the included studies, the reported health outcomes in either diabetes or COPD cases do not seem to be strictly related to the telemedicine consultation technology.

### Associated Behaviors and Attitudes of Patients and HCPs

All the included studies reported on the associated behaviors and attitudes of patients and HCPs during their participation in telemedicine consultations. Patients were generally comfortable, receptive, and satisfied with the approach [39,41,49-51], even if it required the adoption of supplemental at-home monitoring equipment [47]. They appreciated the convenience [45,48,55], time [45,52,56], and cost efficiency [45,52,56] that remote care offered, as well as the ease of access to HCPs’ insights [47,48]. Some also perceived the technology as a welcome source of information [39,47]. In some studies, patients found remote consultations to be as good as in-person visits [40,42,50,56], with some indicating an interest in or preference for remote visits for follow-up appointments [45,51,53,56]. However, some patients raised concerns about cost, treatment, and practical logistics and that clinical examinations were not thorough during internet-based visits [38,43,52,55]. HCPs viewed certain aspects of remote care delivery as helpful, such as having access to patient data, specialist’s insights, and user-friendliness [39,42]. Despite expressing a need to get better acquainted with the remote modality, HCPs appreciated that they could gain comprehensive insights into their patient’s conditions remotely [38,42,44,51]. However, a minority of HCPs reported a reduction in job satisfaction and a diminished sense of connection with their patients [54].

Telemedicine consultations for patients with diabetes in the included studies were met with positive attitudes. Patients with diabetes were more motivated during telemedicine consultations [39,41,49], favored the convenience provided by such means [48,55], and expressed a preference to have follow-up appointments remotely [51]. HCPs appreciated the assistive aspect of telemedicine consultations with patients with diabetes and the insights they could derive remotely [39,51]. While most studies (4/6, 67%) involving patients with diabetes used only video telemedicine consultations [39,41,48,49], 1 (17%) study involved only audio visits [51], and 1 (17%) study included both video and audio visits [55]; the satisfactory experience and positive attitude were shared in all remote modalities among this patient group. Among the included studies that involved COPD cases, patients and HCPs were satisfied with the technology [40,44,47], while a fraction required some familiarization with the setup [38], which is likely reflective of the time of publication (1999). Patients with COPD were receptive to telemedicine consultations [46] and were willing to adopt supplemental technologies for remote monitoring [47]. HCPs further trusted the remote readings [44]. The majority (4/5, 80%) used video consultations [38,40,44,47], and only 1 (20%) study involved audio-only telemedicine consultations [46], but the satisfaction rate was similar in either mode for patients with COPD.

### Potentials for Supplemental Technology Assistance

Among the included studies, potentials for supplemental technology assistance during internet-based consultations were inferred in most studies based on the barriers and concerns reported. Most concerns were related to technical considerations such as malfunctioning computers, audiovisual delay, internet firewall settings, video bandwidth, internet access, and connectivity [41,44,49,51-53]. The need to improve communication and coordination between HCPs for a more efficient workflow during remote consultations was also identified [39,52,56]. Minimizing associated paperwork, data load, resource use, and associated expenses could improve adoption among HCPs [39,40,43,45,47]. Improvements were identified to be associated with the assessment and monitoring of patients remotely, which could involve better image quality or supplemental equipment for specific physical examinations [38,42,52]. Some suggestions included incorporating an educational component for patients into the telemedicine modality, improving the user-friendliness of the platform, and improving translation and auditory feedback [46,53,54].

In particular, for patients with diabetes, improvements for video telemedicine consultations pertained to the web-based workflow [39] and functioning of the setup [41,49], while audio telemedicine consultations could be improved with increased internet connectivity and flexibility of the remote care team [51,55]. For patients with COPD, video consultations would benefit from improved audiovisual quality [38,44], reduced associated costs, and less disruptions to daily routines [40,47]. The identified improvements for audio telemedicine consultations for patients with COPD could involve educational and training components for both patients and HCPs to better use the modality [46].

### Critical Appraisal

No critical appraisal of the included studies was conducted, as it is generally not conducted in scoping reviews [31].

## Discussion

### Principal Findings

This scoping review set out to investigate, in relation to synchronous telemedicine consultations for nonmalignant chronic illnesses, (1) the available evidence on telemedicine consultations to improve health outcomes for patients, (2) the associated behaviors and attitudes of patients and HCPs, and (3) how supplemental technology can assist in remote consultations.

### Study Characteristics

We identified 19 studies that fulfilled the selection criteria, and these were published between 1999 and 2022. Among these, 12 (63%) were conducted before the COVID-19 pandemic in only 2 continents (Europe and North America), while the remaining 7 (36%) publications included studies conducted in Australia and Saudi Arabia. This potentially indicates that the expansion of interest in remote care-related research to more locations might have been precipitated by the shift to adopting such consultation modalities amid the pandemic [57].

The higher number of identified studies relating to diabetes, cardiovascular conditions, and COPD likely results from these specific terms being included in the search strategy. However, recent systematic reviews investigating remote consultations also identified more studies that relate to these conditions [58,59], which could indicate a higher likelihood of remote care being adopted for consulting patients with such conditions, considering their higher prevalence [60-63].

As the majority of the included studies in this scoping review used video consultations, this modality appears to be the most popular one. This is contrary to certain cases such as in the United Kingdom, where remote audio consultations appear to be preferred [64]. Nevertheless, based on the reviewed studies, there appear to be high satisfaction rate among adopters of video consultations [39,40,44,45,48-50,52] and even a preference for video consultations by some HCPs for the nonverbal cues and visual insights that this modality enables [42,53]. While most of the included studies published before the pandemic used video visits [38-42,44,45,47-49], studies that were conducted after the COVID-19 pandemic adopted more heterogeneous means [50-56], with slightly more studies investigating audio-only telemedicine consultations [51,54,56]. The higher diversity in telemedicine consultation means after the pandemic could be attributed to the necessity of using remote care options during the health crisis [18,65]. While significant technological improvements have been achieved since 1999 [66], which is the publication year of this review’s earliest included study, it appears that there has not been much change in terms of the remote modalities adopted, but their means of access has evolved. More novel means of telemedicine consultation such as extended reality and the metaverse, which researchers have considered as potential supplements to telemedicine consultations [20], have not been identified in the included studies. This potentially indicates their nascent aspect, which has not been extensively investigated and could represent a future research topic.

### Reported Health Outcomes for Patients With Chronic Conditions Using Telemedicine Consultations

From the 19 included studies, 14 (74%) reported on the health outcomes of patients using internet-based consultations. The majority experienced positive health outcomes, with improvements directly related to their chronic condition [41,42,44] as well as their mental health [45,48,54]. Improvements were also noticeable regarding treatment control, adherence, and condition management [39,43,51]. Considering these findings from this scoping review, telemedicine consultations indicate the potential to be beneficial not only for patients’ physical health but also for aspects that are, to some extent, tangential to their chronic conditions, improvements to which have been associated with better health outcomes [67,68]. This was highlighted by the recent study by Salah et al [69] investigating the psychological impact of the pandemic on patients with chronic conditions among the Egyptian population. While their findings are limited to the Egyptian population, the researchers found increased rates of anxiety, depression, and stress among this demographic, partly associated with the fear of contracting the SARS-CoV-2 virus, owing to which they subsequently did not attend follow-up consultations. Similar effects were also identified in the review by Dubey et al [70] on the psychosocial impact of COVID-19.

Furthermore, studies identified in this scoping review indicated an improvement in mental health as well as adherence; this indicates the potential for remote care models to address no-shows and anxiety related to in-person visits. This corroborates with the review by Kendzerska et al [71], which identified benefits of telemedicine for chronic disease management to include improved treatment compliance and follow-up rates. Thus, remote care could potentially be associated with empowering patients with chronic conditions to better manage their condition and treatment regime while being beneficial for their physical and mental health.

However, these findings might not be applicable to every case, as a minority of studies did not find telemedicine consultations to add any significant contribution and found that they were comparable to traditional in-person modalities in regard to health outcomes, even if participants were receptive or satisfied with the internet-based care experience [40,46,47,49,50]. Of the 19 included studies, 1 (5%) [46] even identified deteriorating symptoms among patients using telemedicine based on the Clinical COPD Questionnaire scale, which is a validated and reliable measure [72-74]. Such findings are in accord with previous research that highlighted that internet-based care does not offer a “one size fits all” approach [75,76]. Even if a minority of the included studies did not find added health value or even identified adverse health outcomes with telemedicine consultations, such modalities could be better considered as potential options tailored for individual patients’ needs rather than a replacement of traditional face-to-face visits in every case.

Considering the most investigated conditions in the included studies, diabetes and COPD, the need for a tailored telemedicine consultation approach is further highlighted. For instance, patients with diabetes using telemedicine consultations experienced positive health outcomes in most cases [39,41,48,50]. In comparison, positive health outcomes were only identified in a minority of patients with COPD [44], while most experienced no particular health benefits [40,44,46]. These could be attributed to the varying needs of patients with diabetics and patients with COPD, and telemedicine consultations might provide more health benefits for the former group of patients. However, this represents potentials to enhance telemedicine consultations for patients with COPD using supplemental technology aimed at improving outcomes for this specific group.

### Associated Behaviors and Attitudes of Patients and HCPs During Telemedicine Consultations

Each of the 19 included studies provided data relating to the behaviors and attitudes of patients and HCPs during telemedicine consultations. On the basis of these studies, it is notable that even before the pandemic, most patients and HCPs were receptive to and satisfied by technology-mediated consultations [39,41,42,44,45,47-49]. As for the identified studies that were conducted after the pandemic was declared, patients’ and HCPs’ acceptability toward or satisfaction with remote care was high in every case [50-56]. This is in line with previous studies that found a positive attitude toward telemedicine use among patients and HCPs during the pandemic [77,78]. However, such attitudes might have been influenced by the need to adopt remote care technologies imposed by the pandemic [65], rather than these being an optional alternative. Given that the prepandemic studies identified in this scoping review still reported a general receptiveness to and satisfaction with the modality, telemedicine consultations might be considered as a viable option.

From patients’ perspectives, the reported benefits of telemedicine consultations are related to their convenience, time and cost efficiency, ease of access to HCPs’ insights, and informational aspects [39,45,47,48,52,55,56]. Barriers to these aspects have been identified as limitations to health care access for patients with chronic conditions [78-81]. The findings of this scoping review indicate that internet-based modalities can potentially address some of those pertinent barriers, especially considering that some patients viewed internet-based visits as comparable to in-person visits [40,42,50,56]. Considering telemedicine consultations for patients with diabetes, the approach, whether audio or video based, was positively received in each case [39,41,48,49,51,55], and acceptance was also high among patients with COPD [40,44,47], although a minority needed to get accustomed to the video telemedicine consultation setup [38]. This need could be attributed to the outdated equipment used in the study conducted in 1999, and video telemedicine consultation setup has likely improved in recent years.

Nevertheless, such a positive outlook toward telemedicine consultations might not be applicable to every patient, as a minority of the studies included in this review reported that patients were less receptive toward remote care. They expressed negative attitudes toward the modality’s cost, meeting scheduling and logistics, and the thoroughness of clinical examinations [38,43,52,55]. One of the included studies found that racial and ethnic minority groups were less likely to use telemedicine [53]. In addition to reinforcing the notion that internet-based care might not be appropriate in every case [75,76], it reflects the observation of recent studies that highlighted limited adoption by and accessibility of telemedicine for minority groups [82,83]. However, these behaviors toward telemedicine consultations were identified in only a minority of the included studies and could indicate avenues for further investigations.

Regarding the included studies that reported on HCPs’ relevant attitudes and behaviors, the majority indicated positive attitudes and behaviors toward telemedicine consultations. They appreciated the access to patient data and specialists’ insights provided by these modalities and the user-friendliness of the technology [39,42]. They were also satisfied with and trusted the interpretation of patients’ conditions remotely [42,44,51], even if some were unfamiliar with the modality [38,51]. In one of the included studies, HCPs further expressed interest in the continued use of telemedicine after the pandemic [53]. In the case of audio or video telemedicine consultations with patients with diabetes, HCPs were receptive toward the technology [39,41,48,49,51,55]. A similar finding was observed among HCPs involved in telemedicine consultations with patients with COPD [38,40,44,46,47]. Recent studies in different countries have also identified positive attitude from HCPs toward remote care [84-86].

However, those same recent studies have identified limitations to telemedicine from the perspectives of HCPs. For instance, the nationwide survey conducted by Ma et al [85] in China found HCPs’ concerns with the modality to relate to infrastructure, service process, cost, and popularity of such services. The quantitative study conducted by Andronic et al [84] on HCPs’ perception of telemedicine in Romania found limitations to internet-based care associated with the lack of human interaction, dependency on technology, and legislative concerns. The exploratory survey conducted by Ncube et al [86] among HCPs in Botswana identified privacy, associated cost, and required infrastructure and human resource among inhibitors to telemedicine use. While in minority, this scoping review also identified similar behaviors from HCPs [39,54]. They reported mixed perceptions of telemedicine efficiency in regard to the number and length of visits, diminished job satisfaction, and a lowered sense of connection with their patients during telemedicine consultations. This could potentially indicate that some HCPs might have some reservations in regard to remote care use, which could be investigated in future studies to determine factors that could increase satisfaction with telemedicine consultations.

Regarding studies that reported on the HCPs’ attitudes toward different remote care modalities, most were inclined toward video, rather than audio-only, consultation for the audiovisual feedback and nonverbal cues such remote care modalities provide [42,53]. In contrast, a minority showed a more positive attitude toward telephone consultations considering its accessibility for older adults or those with physical impairments [54]. While the low number of included studies that reported on this aspect limits the ability to draw a consensus, this finding might indicate that video consultations might be a preferred option, while audio-only visits could be a consideration based on individual needs and preferences [64].

### Potentials Identified for Supplementing Telemedicine Consultations With Additional Technological Support

On the basis of the included studies’ reported barriers and concerns regarding telemedicine consultation use for patients with noncommunicable chronic conditions, potentials for supplemental technology assistance were inferred. The majority of the included studies reported the need to improve certain technical aspects related to telemedicine consultations. These include malfunctioning computers, audiovisual delay, internet firewall settings, video bandwidth, and internet access and connectivity [41,44,49,51-53]. Considering telemedicine consultations specifically for the most common illnesses investigated, patients with diabetes experienced challenges related to the telemedicine consultation equipment in both video and audio formats [41,49], while audiovisual quality was the major technical issue for patients with COPD using video telemedicine consultation [38,44]. It is somewhat surprising that relatively basic and infrastructural issues were encountered during recent studies when in parallel researchers are encouraging the adoption of more advanced technologies such as the Internet of Things and blockchain in the health care setting [87,88]. This potentially indicates challenges to effectively implementing remote care technologies due to inadequate resources and infrastructure. Such challenges have also been shown to be tied to design aspects, where remote consultation areas in hospitals require a different setting and design from traditional and existing infrastructure [89]. In relation to supplemental technologies providing assistance to address such issues, supplemental technologies providing assistance could be extrapolated from the review by Baker and Stanley [90] of technical aspects of telemedicine technology. The authors suggest that planning for successful telemedicine programs should consider technical aspects and include features such as high-speed and secure internet access, a “telemedicine hub” for conducting remote interactions, patient access software, and access to IT professionals who can address malfunctioning issues.

Some studies included in this review identified the need for better communication and coordination between HCPs [39,52,56]; this could potentially indicate the need to better train and support HCPs in effectively using telehealth. Both patients with diabetes and COPD, the 2 most commonly investigated conditions in the included studies of this review, indicated challenges with the remote workflow [39,47,55], which potentially indicate a need for better training to effectively use remote care. Researchers have also identified a lack of standard recommendations for telemedicine curricula, as well as a limitation in telehealth education and training among HCPs [91-93]. A potential technological assistance could be including within telemedicine software an HCP training module based on a competency-based, outcome-oriented framework, as recommended by Stovel et al [91]. This could be integrated in tandem with an educational component for patients according to their specific condition as well as for telemedicine use, given that this was a suggestion in one of the included studies in this review [46].

In regard to other suggestions for improvement from the included studies, user-friendliness and improving translation and auditory feedback were highlighted [53,54]. While involving a limited number of studies from those included in this review, these observations might indicate the need for a participatory design approach in designing telemedicine services [94]. The review by Fouquet and Miranda [95] further concluded that telemedicine design flaws could be circumvented by involving stakeholders, from HCPs to patients, in the design and implementation of such services. As such, improvements to factors such as user-friendliness and auditory feedback could be undertaken by an iterative development approach such as that highlighted in the Integrate, Design, Assess, and Share framework [96]. During development cycles, supplemental technologies could be integrated to address the identified needs such as translation [54], which is a real-time feature of the internet-based health care company CirrusMD [97]. Other needs identified in the included studies pertained to reducing associated paperwork and data load, resource use, and expenses [39,40,43,45,47]. These could, to some extent, be addressed by integrating speech recognition software for automated medical reporting, which has been identified to reduce administrative burden and lower associated costs [98].

Supplemental technologies can also extend beyond software to include hardware. This could address the need for better assessment and monitoring of patients remotely, as identified in some of the included studies [38,42,52]. Telemedicine consultations could integrate the use of wearable devices appropriate for patients’ conditions with the remote capture of patient data that can be shared with HCPs on the web [99], which the review by de Farias et al [100] found to improve patient care and treatment effectiveness.

**Figure 2:**
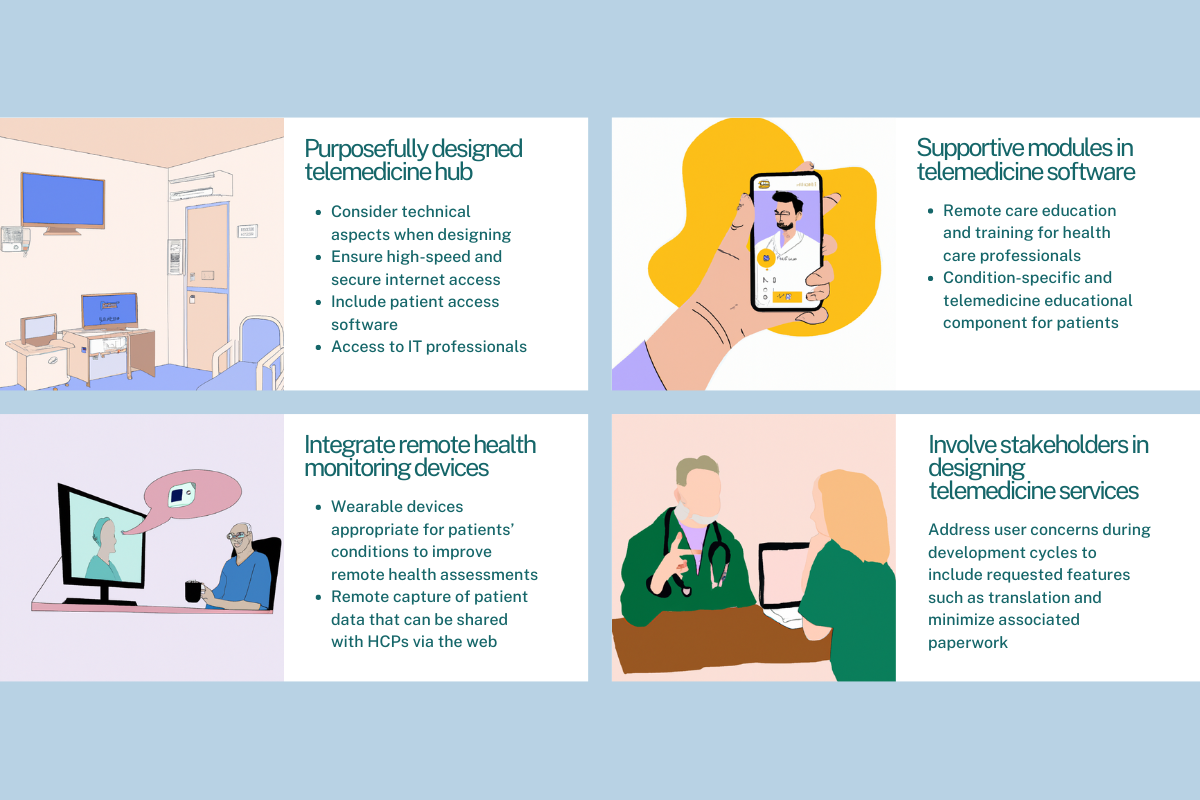
Potentials for technological improvements during internet-based consultations with patients with nonmalignant chronic conditions. (Individual images were generated with the assistance of the generative artificial intelligence tool Dall-E [Open AI].) HCP: health care professional.

To better help visualize the identified potentials for supplemental technology assistance during telemedicine consultations, we summarized and illustrated this scoping review’s findings in this regard in Figure 2.

### Limitations

There are some limitations to the included studies as well as to the scoping review process that require a measure of caution when generalizing the findings. Regarding limitations of the included sources of evidence, while the average participant size was 401, this number was disproportionately affected by 1 study that included >5000 participants [53], which can influence the research findings [101]. Some measure of discretion is advised regarding the generalizability of the findings of health outcomes, as only a minority of the reviewed studies included patients through a follow-up period [39,41,43,46] or involved control groups [40,42,44,46,49,50]. Only 1 of the reviewed studies included both follow-ups and control groups [46]. Despite the difference in study design and scope, including follow-ups and control groups are recommended practices in telemedicine research to gain more comprehensive insights into outcomes [102,103]. As a minority of included studies did not specify the conditions involved [52,53], the results need to be interpreted with a layer of caution.

While we followed recommended guidelines for conducting this scoping review, some limitations persist. Most prominently, the number of included studies can limit the generalizability of the findings. This is due to the filtration process, which, for example, excluded studies that did not involve synchronous consultations or studies that did not involve interactions with HCPs, and insights from excluded studies were not considered. However, the inclusion criteria were designed by the research team to adhere to the research aims. In addition, this review considered extracted data and synthesized insights from different chronic conditions and did not consider individual conditions in relation to the review’s objectives. Therefore, caution is advised when extrapolating the findings to specific conditions.

### Conclusions

This scoping review provided important insights into the health outcomes, attitudes, and potentials for technological improvements in relation to using telemedicine consultations for patients with nonmalignant chronic illnesses. On the basis of the reviewed studies, patients with noncommunicable chronic conditions who use such remote care modalities are mostly patients with COPD or diabetes, although remote means is also used for providing care for people with other chronic conditions. According to our findings, patients with chronic conditions generally experience positive health outcomes that are either directly or indirectly related to their ailment. However, sustained improvements cannot be confidently established due to limited follow-up data, and positive health outcomes might depend on the condition, with patients with diabetes mostly experiencing positive outcomes, while patients with COPD generally did not find added health benefits. Nevertheless, our findings also identified that telemedicine consultations could be considered as an empowering tool for patients with chronic conditions to assist them in better managing their condition and treatment regime.

This review indicates a general receptiveness toward telemedicine consultations based on the positive related attitudes and behaviors when such consultations are an option. Most patients favor the convenience, increased access to care, expert insights, and health education that remote care options provide. HCPs tend to be satisfied with the interpretation of patients’ conditions remotely and have an apparent preference for video-based modalities considering the audiovisual feedback and nonverbal cues that they provide.

Potentials for supplemental technological assistance in internet-based consultations have also been identified from the included studies. These mostly relate to technical challenges due to inadequate resources and infrastructure and could be addressed during the planning stages of telemedicine programs. Telemedicine software could also integrate telehealth education and training modules for HCPs and patients to address the respective challenges they experience. It could further be recommended that the design of telemedicine services adopts a participatory aspect with stakeholders.

However, this review highlights that telemedicine consultations might not be the most appropriate option for a minority of cases. In relation to remote care, some patients with nonmalignant chronic conditions might not experience positive health outcomes, might not find the modality accessible, or might not be satisfied by the modality. A portion of HCPs might hold reservations to using telehealth due to a diminished sense of job satisfaction and a reduced sense of connection with their patients during telemedicine consultations. This reinforces the notion that telemedicine consultations might not represent a “one size fits all” approach and should be better considered as an option that can be tailored based on specific needs rather than a complete replacement of in-person visits.

Nevertheless, considering the general receptiveness and positive health outcomes associated with such modalities, telemedicine consultations might be considered as viable options to be recommended to patients with noncommunicable chronic conditions and their HCPs, with the potential to improve their perception of the modality with supplemental technology.

## Appendix

Multimedia Appendix 1 Search term combination used in CINAHL Complete (via EBSCOhost).

Multimedia Appendix 2 Extracted data from included studies.

Multimedia Appendix 3 PRISMA-ScR Checklist.

## Abbreviations

CCC: chronic cardiovascular condition
COPD: chronic obstructive pulmonary disease
HCP: health care professional
PRISMA: Preferred Reporting Items for Systematic Reviews and Meta-Analyses
PRISMA-ScR: Preferred Reporting Items for Systematic Reviews and Meta-Analyses extension for Scoping Reviews
