# Supplementary material for: Evaluation of Telemedicine Consultations Using Health Outcomes and User Attitudes and Experiences: Scoping Review
Source: J Med Internet Res. 2024 Jul 9;26:e53266. doi: 10.2196/53266 (PMC11267102; doi:10.2196/53266)
Supplement: Multimedia Appendix 1 [file jmir_v26i1e53266_app1.docx]

### Appendix 1: Search terms combination used in in CINAHL Complete (via EBSCOhost)

The final search results (labelled as S29 below) were obtained following a successive combination of terms including the scoping review’s initial search terms and suggested subject terms from the CINAHL Complete database.

S1: "virtual consultation"

S2: (MH "Remote Consultation") OR "remote consultation"

S3: (MH "Telemedicine+") OR (MH "Telehealth+")

S4: (MH "Telehealth+") OR "telehealth" OR (MH "Ambulatory Care Nursing")

S5: "metaverse"

S6: (MH "Virtual Reality+") OR "virtual reality" OR (MH "Virtual Reality Exposure Therapy")

S7: (MH "Augmented Reality") OR "augmented reality"

S8: "mixed reality"

S9: "extended reality"

S10: S1 OR S2 OR S3 OR S4 OR S5 OR S6 OR S7 OR S8 OR S9

S11: (MH "Chronic Disease+") OR "chronic conditions"

S12: "chronic illnesses"

S13: (MH "Chronic Disease+") OR "chronic disease" OR (MH "Noncommunicable Diseases")

S14: (MH "Diabetes Mellitus+") OR "diabetes mellitus" OR (MH "Diabetes Mellitus, Type 2") OR (MH "Diabetes Mellitus, Type 1+") OR (MH "Diabetes Mellitus, Gestational")

S15: (MH "Pulmonary Disease, Chronic Obstructive+") OR "chronic respiratory illnesses"

S16: (MH "Cardiovascular Diseases+") OR "cardiovascular conditions"

S17: (MH "Obesity+") OR "obesity" OR (MH "Obesity, Maternal") OR (MH "Obesity, Morbid")

S18: (MH "Pulmonary Disease, Chronic Obstructive+") OR (MH "Asthma-Chronic Obstructive Pulmonary Disease Overlap Syndrome") OR (MH "Lung Diseases, Obstructive+") OR "COPD"

S19: S11 OR S12 OR S13 OR S14 OR S15 OR S16 OR S17 OR S18

S20: (MH "Physical Therapist Attitudes") OR (MH "Physician Assistant Attitudes") OR (MH "Respiratory Therapist Attitudes") OR (MH "Attitude of Health Personnel+") OR (MH "Psychotherapist Attitudes") OR "attitudes" OR (MH "Physician Attitudes") OR (MH "Consumer Attitudes") OR (MH "Attitude to Illness+") OR (MH "Patient Attitudes") OR (MH "Social Attitudes")

S21: "experiences"

S22: "engagement"

S23: "behaviours" OR (MH "Social Behavior+")

S24: (MH "Intention") OR "intentions"

S25: (MH "Motivation+") OR "motivations"

S26: (MH "Psychology+") OR "psychology"

S27: (MH "Health Services Accessibility+") OR (MH "Personal Boundaries+") OR (MH "Communication Barriers+") OR "barriers"

S28: S20 OR S21 OR S22 OR S23 OR S24 OR S25 OR S26 OR S27

S29: S10 AND S19 AND S28
