# Supplementary material for: Evaluation of Telemedicine Consultations Using Health Outcomes and User Attitudes and Experiences: Scoping Review
Source: J Med Internet Res. 2024 Jul 9;26:e53266. doi: 10.2196/53266 (PMC11267102; doi:10.2196/53266)
Supplement: Multimedia Appendix 2 [file jmir_v26i1e53266_app2.docx]

### Appendix 2: Extracted data from included studies

***List of abbreviations used in Appendix 2: CHF - congestive heart failure, CKD - chronic kidney disease, COPD - chronic obstructive pulmonary disease, HCP - healthcare professional, PCP - primary care provider, telemedicine video consultations (TVC)***

| **Author/**  **Year/**  **Country** | **Study design and aim(s)** | **Chronic condition(s) involved** | **Virtual consultation modality employed** | **Reported health outcomes** | **Related attitudes and behaviours** | **Potentials for technological improvement** |
| --- | --- | --- | --- | --- | --- | --- |
| Mair  1999  UK  [38] | Mixed-methods pilot study to investigate the feasibility of using telecommunications technology to assist in the support of acutely ill patients with COPD exacerbations at home | COPD | Real-time, interactive video-phone visits | No health outcomes reported | Patients and healthcare professionals (HCPs) required getting accustomed to the setup | Improve image quality to enable assessment of respiratory rate/cyanosis |
| Tudiver  2007  USA  [39] | Phone survey interview to evaluate primary care provider’s (PCP) perceptions of remote diabetes care delivery | Type 2 Diabetes | Home telemedicine units for patient intervention with videoconferencing service and remote glucose and blood pressure monitoring | Increased control, confidence, compliance and motivation in managing diabetes among patients. | Positivity towards acceptability of telemedicine  **Patients:** Experienced positive health education; felt more motivated  **PCPs:** Found access to more patient health data helpful; appreciated specialist consultation; better quality of patient encounter  Mixed perception of telemedicine efficiency (number and length of visits) | Improve communication between HCPs; increase patient adoption of telemedicine; reduce paperwork and data volume; clarify management changes in visit notes |
| Whitten  2007  USA  [40] | Mixed method study to evaluate the  use of home telehealth for patients with chronic obstructive pulmonary disease (COPD) and/or congestive heart failure (CHF) | COPD and/or CHF | Home video conferencing equipment with peripheral devices for monitoring vital signs | No significant effects/equivalent to traditional services | Satisfied with care delivery and technology | Telehealth may provide added value if the associated costs are reduced, even if the outcomes are similar |
| Trief  2008  USA  [41] | Qualitative study to describe the lived experiences of elderly diabetics involved in a telemedicine management intervention | Diabetes | Home telemedicine unit with videoconferencing feature and the ability to uploading blood pressure and glucose readings | Positive physical health changes (including glucose readings/insulin use, eating habits, activity level)  Some emotional benefit experienced by a segment of participants | HCP encouragement can positively influence participation  Participation motivated by desire to feel and be healthier | Some frustrations with operating the technology and computer functioning, which were resolved with technical assistance or new hardware |
| Nilsson  2009  Sweden  [42] | Quantitative evaluation of the feasibility  and quality of uncomplicated hypertension care in rural areas, with comparison between telemedicine and face-to-face consultations | Primary hypertension | Patient: videoconference system  (880 NPP, Tandberg), with a control centre and a 81-cm monitor  Physician: laptop, (T42, IBM), with a webcam | Patients treated via telemedicine had a higher probability of improving their blood pressure | Malfunctioning equipment was the main cause for not employing telemedicine  **Patients:** Most found telemedicine to be as good as in-person GP meeting  **Nurses:** Found videoconferencing to provide comprehensive patient evaluation; allowed them to work more independently  **Physicians:** User-friendly and practical equipment that improves cooperation with nurse and patient  Audio and visual feedback from patient preferred over telephone consultation | Equipment not found to be useful for remote lung examination |
| Cook  2010  USA  [43] | Quantitative evaluation to assess telephone-delivered nurse counselling to address cognitive and emotional barriers to medication adherence in ulcerative colitis | Ulcerative colitis | Telephone call between nurses and patients, involving motivational interviewing and cognitive-behavioural techniques | Treatment adherence up to 6 months was higher than the expected rate | High attrition rate (49%) but unrelated to adherence  High adherence among patients followed  Non-starting participants concerned about cost, adverse drug effects and treatment logistics  Treatment discontinuation due to negative beliefs about treatment and breakthrough symptoms | Concerns around cost, and treatment logistics |
| Sorknæs  2010  Denmark  [44] | Mixed method interventional study to investigate the effect on early readmissions of telemedicine video consultations (TVC) for COPD patients in their homes after a hospital discharge | COPD | Video consultations between patients and nurses with telemedicine equipment composed of a computer with a web camera, a microphone and measurement equipment | 10-14% reduction in readmission risk for TCV patients | High satisfaction rate with patients and nurses  Nurses trusted measurement collected from consultations | Minimise audiovisual delay (due to satellite use) |
| Campbell  2011  Canada  [45] | Mixed method feasibility study to determine whether telemedicine was a feasible way to provide health care and support to rural communities and to assess the level of satisfaction among patients and HCPs | Chronic kidney disease (CKD) | Videoconferencing unit and an electronic stethoscope | Patients experienced less stress than in-person visits | High level of satisfaction amongst all participants, who expressed interest to continue using telemedicine  Patients appreciated the ease of access to care, and time and cost savings | Improve flow of paperwork and access to patient information. |
| Berkhof  2014  The Netherlands  [46] | Quantitative pilot study to determine the effects of telemedicine on health-care utilisation and health status of COPD patients | COPD | Structured phone call with registered nurse | No improvement of health status among telemedicine group  Improved Clinical COPD Questionnaire symptom domain score for control group but deteriorated for telemedicine group | Use of audio telephone consultations led to little loss to follow up | Include educational/pulmonary rehabilitation/training component to drive a more successful telemedicine model  Minimise resource use |
| Mathar  2015  Denmark  [47] | Qualitative interviews investigating the experiences and preferences of COPD patients in relation to discharge from hospital with televideo consultations | COPD | Televideo consultations | No special value attributed to intervention in tackling health-related issues | Patients are receptive of the technology and to adopt at-home monitoring equipment  Technology is perceived as a good source of information, a welcome source of distraction, and HCP access provides a sense of control | None interested in future televideo consultations following hospital discharge due to interference with daily routines, cost considerations and unwanted obligations |
| Raymond  2016  USA  [48] | Pilot study to investigate the feasibility and acceptability of a telemedicine intervention | Type 1 Diabetes | Vidyo web-conferencing software | Potentials identified to improve mental health, diabetes education, and condition management-related problem solving | Satisfactory experience given improved convenience, ease of access to HCP insights and time efficiency | N/A |
| Reid  2018  USA  [49] | Quantitative cohort pilot study to assess feasibility and acceptability, care retention and follow-up rates, patient satisfaction, and adherence to guidelines in a telemedicine intervention | Type 1 Diabetes | Vidyo web-conferencing software | No significant change in HbA1c values in either telemedicine or control group | Higher engagement, adherence and frequency of clinical visits in telemedicine group  Higher satisfaction rates among telemedicine group regarding care experiences and technology | Barrier to access encountered due to institutional Internet firewall settings |
| Lambooy  2021  Australia  [50] | Quantitative case-controlled longitudinal observational cohort study to assess the feasibility, sustainability, and clinical outcomes of telehealth videoconferencing (TVC) for this patient population. | Chronic kidney disease | HCPs: desktop computer with specific telehealth software  Patients: desktop computer, tablet, or smartphone with appropriate software and a dial-up code to access appointment | No between-group differences during follow-up for 2 years in kidney function, blood pressure, mortality, or hospitalisation | Very high patient satisfaction with the care provided  TVC was comparable to standard care  TVC uptake reduced over time | Reduce concerns with technology and reminder that TVC is an option |
| Magliah  2021  Saudi Arabia  [51] | Cross-sectional quantitative analysis of online surveys to evaluate patient perception of virtual phone clinics during COVID-19 pandemic | Type 1 Diabetes | Audio phone call | Improved diabetes control and reduced risk of contracting COVID-19 | **Patients:**  Satisfied with modality and perceived it as positive  Majority expressed preference to attend follow-up appointments through phone consultations  **Physicians:**  Good understanding of patients’ condition via phone despite limited experience with the modality | Improve internet access to increase participation in survey |
| Singh  2021  USA  [52] | Online surveys with quantitative analyses to determine the limitations of telehealth accessibility, patient satisfaction with telehealth relative to in-person visits, and the perceived advantages and disadvantages to telehealth | Cardiac conditions (unspecified) | Any telehealth modality | None reported | High level of satisfaction with telehealth across modalities; time and cost savings seen as advantageous  Clinical exam not perceived as thorough by some patients | Improve internet connectivity, improve coordination of telehealth appointments; incorporate remote patient monitoring solutions |
| Balut  2022  USA  [53] | Mixed methods study to understand telemedicine use before and after COVID-19, and identify relevant barriers and facilitators to its implementation | Cardiology (unspecified) | Synchronous telephone or video visits | None reported | **Patients:**  Minority groups less likely to use telemedicine  Preference of telephone over video visits due to lack of equipment or video bandwidth and challenges with platform  **Physicians:**  Preferred video to phone visits for non-verbal cues and visual feedback of patients’ living situation  Willingness to keep using telemedicine after the pandemic | Improve access to video consultations with equipments, video bandwidth and user-friendliness of video conferencing platform |
| Heyck Lee  2022  Canada  [54] | Quantitative cross-sectional observational survey to evaluate patient and physician perspectives on the key advantages and disadvantages of telephone consultations in a nephrology out-patient clinic setting during COVID-19 | Chronic kidney disease | Telephone consultation | Reduced anxiety related to contracting COVID-19 by travelling to clinic | No major technical challenges or confidentiality concerns  **Patients:**  Comfortable with modality which was also preferred for being time- and cost-efficient  Low preference for video conferences (9%) while some preferred in-person visits for interpersonal connection and clarity of physical changes  **Physicians:**  Found telephone visits advantageous for its accessibility for elderly or people with physical disabilities  Less job satisfaction and sense of connection with patients and identified barriers included language barriers, hearing impairment, and inability to physically examine patients | Improve translation and auditory feedback to increase patient access to audio consultations |
| Hitchcock  2022  UK  [55] | Service evaluation with online questionnaire and telephone interviews to understand patients’ perspectives on virtual appointments, the accessibility of the service and inform its development | Diabetes | Video consultation or telephone call | Not reported | Appreciated at-home convenience  Barriers identified: practical (travel, work, and technical issues); psychological (forgetting, anxiety around appointments, and mental health); and patients’ experiences of relationships with the diabetes team | Increase flexibility and access to service/team |
| Lohnberg  2022  USA  [56] | Quantitative programme evaluation with phone surveys to report on the transition to a remote modality for a weight management programme during COVID-19 | Obesity | Telephone care for patients  Zoom/Microsoft Teams for interdisciplinary team | Not reported | About half found telephone visits to be as good as, and about one‐third thought telephone visits were inferior to in-person visits.  Half indicated preference for remote visits after pandemic  Time and cost efficiency of remote care compared to in-person care favoured | Video meetings for interdisciplinary team cumbersome due to hardware and software requirements; and require adequate training |
